# How do patients with severe mental diagnosis cope in everyday life - a qualitative study comparing patients’ experiences of self-referral inpatient treatment with treatment as usual?

**DOI:** 10.1186/1472-6963-14-347

**Published:** 2014-08-15

**Authors:** Marit B Rise, Gretha H Evensen, Inger Elise O Moljord, Marit Rø, Dagfinn Bjørgen, Lasse Eriksen

**Affiliations:** Department of Public Health and General Practice, Norwegian University of Science and Technology, Trondheim, Norway; Norwegian Resource Center for Community Mental Health, Trondheim, Norway; Nidaros Community Mental Health Centre, St. Olav’s University Hospital, Trondheim, Norway; Resource Centre for Service User Experience and Service Development, Trondheim, Norway; Department of Neuroscience, Norwegian University of Science and Technology, Trondheim, Norway

**Keywords:** Mental health services, Self-referral, Mental illness, Psychiatry, Psychosis, Bipolar disorder

## Abstract

**Background:**

Several hospitals in Norway provide short self-referral inpatient treatment to patients with severe mental diagnosis. No studies have compared the experiences of patients who have had the opportunity to self-refer to inpatient treatment with patients who have received treatment as usual. This qualitative study was nested within a randomised controlled trial investigating the effect of self-referral to inpatient treatment. The aim was to explore how patients with severe mental diagnosis coped four months after signing a contract for self-referral, as compared to patients receiving treatment as usual.

**Methods:**

Data was collected using qualitative individual interviews with patients with severe mental diagnosis, conducted four months after being randomised either to a contract for self-referral (intervention group) or to treatment as usual (control group).

**Results:**

Twenty-five patients participated in interviews - 11 from the intervention group and 14 from the control group. Results four months after randomisation showed that patients with a contract for self-referral appeared to have more confidence in strategies to cope with mental illness and to apply more active cognitive strategies. Patients with a contract also expressed less resignation, hopelessness and powerlessness than patients without a contract. In addition, patients with a contract seemed to be closer to the ideal of living a "normal" life and being a "normal" person.

**Conclusion:**

The results indicate that the patients who had a contract for self-referral had come further in the recovery process and should possibly be better off during treatment.

**Electronic supplementary material:**

The online version of this article (doi:10.1186/1472-6963-14-347) contains supplementary material, which is available to authorized users.

## Background

Mental health services have changed considerably during the last decades. In Norway, an increasing number of mental health service users live outside institutions and receive health care from community health services, ambulatory service teams or outpatient treatment in community mental health centers [1]. The focus has also shifted from perceiving severe mental illness as a permanent diagnosis towards focusing on what facilitates full or partial recovery [2]. Recovery is described as a process where the illness’s negative impact is minimized by focusing on the patient’s strengths and interests [3]. This shift has also moved the delivery of mental health services towards less institutional treatment and more integration in the patient’s community [4].

Ambulatory health services and outpatient treatment are considered to be important and cost-efficient means of strengthening the service user’s independence and responsibility for the treatment [4]. Outreach mental health services via community teams are widespread in the US and in Europe, especially for severe and chronic mental problems such as psychosis and schizophrenia [5], and are also provided in crisis [6]. It has been argued that institutional hospitalization should be reduced, and that when inpatient treatment is necessary, restraints should be minimized and patient control and self-determination strengthened [4].

Health service users have described that services at home, for instance from ambulatory acute treatment teams, have increased feelings of being in control and being seen and heard, making the users feel more responsible for the treatment [7]. Several qualitative studies have shown that the experience of control, autonomy and power to make choices is connected to improved quality of life [8], thereby underlining the importance of such experiences.

Although crisis resolution is increasingly provided in the service user’s home [6], periodically inpatient treatment is still necessary for patients with severe mental illness [9]. Mental health service users have emphasized that services should be flexible and provide safe and predictable care in phases with strong symptoms, as well as facilitating the user’s responsibility and ability to cope in phases with fewer symptoms [10]. This emphasis suggests a combination of services such as home-based care to strengthen coping in good phases, and, periodically, inpatient care in phases with more symptoms. Even when inpatient treatment is necessary, it is essential to encourage and maintain the patient’s control and responsibility. One way to achieve this is to give patients the opportunity to self-refer to inpatient treatment.

The intention behind self-referral inpatient treatment is, in addition to reduce health service costs, to empower the service user and thereby improve symptoms as well as increase responsibility, coping, and quality of life. Giving service users the opportunity to self-refer provides the users with decision-making power during severe mental illness, and should therefore transfer responsibility and power to the service users. Several mental health hospitals in Norway provide self-referral to short inpatient treatment for some service users [11–14]. Patients can refer themselves to short inpatient treatment when they feel that this is necessary. Such hospitalization is meant to be in line with the service user’s wishes and needs, and the service user is considered the best judge of when inpatient treatment is necessary. The proposed effects of self-referral inpatient treatment include reduced numbers of inpatient hospitalizations, fewer acute hospitalizations in crisis, more appropriate use of inpatient treatment, improved quality of life, and improved coping [11–14]. So far, there is no robust evidence for these effects. Internal evaluations have shown that the total amount of inpatient treatment can be reduced when service users have the opportunity to self-refer. One of these evaluations (published in Norway) indicated that the opportunity to self-refer for up to five days of inpatient treatment increased the total frequency of hospitalizations but decreased the number of hospitalization days by over 30%. In addition, the total amount of coerced hospitalizations was reduced by 50% six months after signing the contract [13]. Service users have also reported satisfaction with the opportunity to self-refer and have emphasized that services became more predictable, increased the feeling of safety and reduced symptoms [11]. Moreover, patients have reported that self-referral gave them more choices and a stronger feeling of control and autonomy [11, 12]

A randomised controlled study is currently investigating the effect of self-referral inpatient treatment in a community health center in Mid-Norway. This paper describes a qualitative study nested into this randomised controlled trial. The aim was to explore the patients’ experiences with the intervention. It is reasonable to believe that self-referral to inpatient treatment will influence the patients’ experience of control and autonomy. Exploring the patients’ experiences would provide valuable knowledge about a new and growing treatment approach in mental health services. No studies thus far have compared the experiences of patients who have the opportunity to self-refer with patients who receive treatment as usual.

### Aim

The aim of the study was to compare the experiences of service users who had signed a contract for self-referral to inpatient treatment with the experiences of users who received treatment as usual, four months after randomization.

## Methods

### Design and setting

This was a qualitative interview study involving patients at a community mental health center in Mid-Norway. The study was nested into a randomised controlled trial (RCT) investigating the effect of a contract for self-referral inpatient treatment on the numbers of hospitalizations and hospitalization days, mental health symptoms and functioning, quality of life and coping (trial registration no. NCT01133587). Patients randomised to the intervention group in the RCT signed a contract with the hospital. This contract gave the patients authority to refer themselves to up to five days of inpatient hospitalization when they felt that such treatment was necessary. In addition to any hospitalizations initiated by health personnel, patients with a contract could thus admit themselves without contacting their doctor, duty doctor or emergency department. The contract was an internal written agreement between the patient and the mental health center and had legal status as such. The contract was an addition to the regular treatment offered and did not replace the patients’ regular treatment plan. Patients randomised to the control group received treatment as usual and were offered a contract for self-referral after taking part in the RCT.

### Participants and recruitment

Inclusion criteria for the RCT were a diagnosis of psychosis or bipolar disorders with or without abuse problems. All participants were in need of long-term treatment in the hospital unit and the psychiatric community service. Health professionals with treatment responsibilities suggested participants for the randomized trial. All participants in the RCT were asked to participate in individual qualitative interviews.

### Data collection

Twenty-five patients were interviewed four months after randomisation - 11 in the intervention group and 14 in the control group. The interviews were conducted between November 2010 and December 2012, either in the hospital or in the patient’s home. Three of the authors (MR, IEOM and GHE) conducted the interviews. All are psychiatric nurses and have extensive clinical experience. A research associate with extensive user experience participated in some of the interviews as well. The interviews lasted between 20 and 60 minutes. Themes in the interviews were taken from a pre-defined, semi-structured interview guide with open-ended questions (Additional file 1). The main questions in the interviews related to how the patients experienced their current mental health problems and everyday life. Typical questions were: "How is your life at present?" and "How is it for you when you are feeling ill?" All participants were invited to elaborate on what they did to prevent symptoms and maintain stability, on their experiences of previous and current symptoms and on current or previous experiences with mental health services. To avoid influencing the participants during the RCT and to ask the same questions of patients in both groups, no participants were asked directly about their experiences with the interventions. The interview questions were therefore general and focused on the participants’ overall situation at the time. The interventions thus became topics in the interviews only if the participants brought it up themselves. All interviews were transcribed verbatim, although redundant words and pauses were deleted and local dialect was translated to written language. The quotes used in the result section were translated into English by the first author (MBR) and verified by the rest of the author group.

### Analysis

The analysis was conducted by a group consisting of a clinical psychologist, a researcher with extensive user experience, three psychiatric nurses, and an academic researcher on public health. The aim of this study emerged during an initial reading of the transcripts with a different research question in mind and with the intention to analyze the interviews across group allocation. Since there seemed to be several differences between the descriptions of the participants in the two groups, we chose to pursue these differences further. The authors were at that point already aware of the participants’ group allocation, and we did not add new or "blinded" authors to the group. The interviews with patients from the control group (treatment as usual) were read and coded first, from which a list of codes (meaning units) and main themes were made. The coding process was conducted during several meetings in the author group. Then, the interviews with the patients from the intervention group (contract for self-referral) were coded. The code list and main themes from these interviews were compared with the codes and themes from the control patients. The main themes common for both groups were:

**Main themes from interviews:**

Experiences of disease and symptoms

Strategies of coping

- Shielding
- Structure (food, sleep, medication)
- Distractions
- Social life
- Thinking strategies

Identity as a person with an illness

Feeling safe

Relationships (family, friends)

Work and studies

Dignity and power

Values in life

A normal life

Four differences between the intervention group and the control group were found, which were then outlined and discussed in the author group. After the group analysis and discussion the first author (MBR) recoded all interviews and cross-checked the results with the transcripts to challenge the findings and look for different interpretations. The process confirmed the findings from the group’s analysis. Quotes from the interviews were chosen to illustrate and substantiate the results. Quotes used in the result presentation are marked with the speaker’s gender and study group (intervention or control). The final results were discussed and approved by the author group during the writing process.

### Ethics

The regional committee for medical ethics in Central Norway approved of the study, which was registered with the Norwegian Data Inspectorate. All participants were given verbal and written information about the study, and signed a consent form before taking part in interviews. All participants were given the opportunity to make contact with the staff at the department after the interview.

## Results

Twenty-five patients participated in this study, 11 had a contract for self-referral to treatment (intervention group) and 14 received treatment as usual (control group). Nine of the participants were female. The mean age was 41 years (range 21-60 years). The participants had one or several diagnoses, including psychosis, bipolar disorders and substance use. Eight of the participants had a job or were studying. The sample is described in Table 1.

**Table 1:** **Characteristics of participants**

Variables		Total sample	Intervention (contract for self-referral)	Control (treatment as usual)
No. of participants		25	11	14
Gender	Females	9	6	3
Age (mean (range))		41 (21-60)	44 (31- 58)	33 (21-60)
Diagnosis (some have more than one)	Psychosis (ICD-10; F 20-29)	19	7	12
	Bipolar disorders (ICD-10; F 30-39)	6	4	2
	Substance use (ICD-10; F 10-19)	6	3	3
	Others (ICD-10; F 60-61)	2	1	1
No. of years since receiving diagnosis (mean (range))		9 (2-23)	10 (4-19)	8 (2-23)
Employment	Paid work	3	2	1
	Disability benefits	14	7	7
	Sick leave	3	-	3
Studies	Studies or serious plans to study	5	2	3

The results present four aspects where the experiences of patients with a contract for self-referral appeared different than the experiences of patients who received treatment as usual. Patients with a contract expressed stronger confidence in strategies to cope with mental illness and seemed to apply more active cognitive strategies. Patients with a contract also had fewer expressions of resignation, hopelessness and powerlessness than patients who received treatment as usual. In addition, patients with a contract appeared to be closer to the ideal of living a "normal" life and being a "normal" person. The differences between the groups are described in light of what were common for all participants.

### Stronger confidence in coping strategies

All participants described several strategies to prevent and cope with symptom aggravation. They emphasized the importance of living a structured life with regular meals, sleep and medication, as well as avoiding smoking, excessive alcohol and drugs. Participants also talked about strategies to shield and distract themselves. Common shielding strategies were to be quiet, to be alone, to relax, and to avoid an excess of impressions, such as too many people or TV programs. Common distraction strategies were to watch TV, play computer games, read books, exercise and work, all of which could help curb anxiety, voices and visions. The participants also described that being with people or animals could both act as distraction, and provide meaningful relationships and experiences. These interactions could help to prevent and diminish symptoms. Overall, the participants described that a balance between activity and rest was necessary to cope with the mental illness.

*P: I have discovered that the more I talk to other people… the more stable I am. It is a sort of self-treatment or self-medication. To actively seek contact with other people every day. Some days, when I can’t get in touch with anybody I walk down to the city and look at people. I do that because I don’t want to be alone. That’s a bad idea. Other days I have a strong need to be alone. Then I withdraw, turn off the phone and don’t meet a single person during the whole day. I sit all day and watch TV-series or DVDs or play Playstation games or read a book. And these days come and go. There is no pattern – some days are like this and some days are like that.*

*[…] I: So books help you?*

*P: Yes, it is good to have something where things happen. […] When I read I disappear a little. I disappear a little from this world. I just dream myself into the book. It is very relaxing. And it is a good way to ignore the things I don’t want to think about… things that are stressful to think about.*

(Male, Control group)

Although all participants identified coping strategies, there were some differences between the ways patients with and without a contract for self-referral described them. Patients with a contract voiced stronger self-confidence in applying coping strategies, both to prevent symptoms and to handle symptom aggravation. These patients also expressed greater confidence in keeping a good balance between activity and rest. Their descriptions appeared to include more experiences in applying and mastering various coping strategies. While patients without a contract described fairly common strategies to cope and prevent symptoms, patients with a contract appeared to have stronger and more independent creativity when describing how they prevented and coped with poorer phases. Some of the patients with a contract also described new and different ways to cope with their illness. Some of these strategies contradicted the advice usually given by professionals, such as preferring to be alone instead of having an extended social life. One patient, for example, expressed a deep satisfaction with having a certain distance from other people:

*P: I like to eat alone. I do. I have discovered that I like to do some things on my own. […]*

*I have two friends at present and we have a good distance… a good distance. We text each other and meet maybe once a week, and I really appreciate that.*

*I: The friendship?*

*P: Yes, the distance when we meet so rarely. We are in touch on the phone… to state that we are fine. […] I read somewhere that friendship is what you think about the other person […] having a presence even though they are not present.*

(Male, Intervention group)

### Cognitive strategies to influence thinking patterns

All participants reported that they experienced anxiety, mood swings and negative thoughts. Almost all participants also talked about disturbing voices and visions, describing cognitive techniques to cope with these symptoms. In addition, all patients relayed that their thinking was associated with control over their situation. Despite these commonalities, comparison showed that the participants used these techniques differently. Patients without a contract talked mostly about cognitive strategies as a means of adapting to the illness and helping them to do their best. They used words such as "observing", "understanding", and "recognizing" symptoms and emphasized accepting the illness. Their main focus centered on being conscious about their thoughts and views and on achieving more knowledge about the nature of the symptoms in order to decrease anxiety when those symptoms rose. One participant described cognitive strategies such as observing and recording the physical surroundings during strong anxiety and claustrophobia:

*P: I am supposed to observe and notice details in rooms and such… when these things happen. I don’t know if it has helped so much. It has become worse and worse. And the feeling is very strong when I am in the midst of it. I almost have to vomit. It affects me physically.*

(Female, Control group)

Meanwhile, patients with a contract talked less about recognizing, understanding and accepting. Instead, they used words such as "power", "control" and "strength". They also displayed stronger ability to influence their thinking patterns through the use of cognitive strategies. Furthermore, these patients talked more about being active and working with themselves in order to grow, to break barriers and to take opportunities. Some of them questioned if the voices and views were true, or whether there were other ways to interpret and understand them:

*P: […] I used to think that everybody knew more than me about certain things. I don’t anymore. […] It’s just confusing… and that’s a difficult state. You become unsure of yourself and that’s heavy. It’s not that I know so much, but what do other people know? […] I read for instance in a book about what happens after death. And I said to myself: How can he [the author] know more about that than me? Has he been there - or is he sitting on this side and imagining what happens afterwards? These simple things… I use tactics like these. Then it is not so bad. […]*

*It still annoys me when someone comes and tells me I can never get rid of it [the illness]. It will probably never be completely over, but… […] There are limits to what I don’t know [laughs]. It is important to believe in the experiences you have. Others might have similar or opposite experiences, but they are not necessarily stronger or more correct.*

(Male, Intervention group)

The results also indicated that patients with a contract had more experience and more control over the cognitive techniques, as well as a deeper understanding of the relationship between thoughts and feelings. They seemed to be more focused on the possibility of choosing cognitive strategies to influence their thinking. While patients without a contract described some efforts to manage being in a painful situation, patients with a contract presented a wider variety of techniques to influence their mental state. This enabled them to change their ways of thinking and thus influenced their overall situation.

*P: Nowadays, I read a lot of self-help books. I used to be very skeptical… they had very simple solutions, but now I find them developing.*

*I: What do you get from them?*

*P: I guess they speak to my own problems. You work with yourself while you read. For example to shut out the past… that is described in all self-help books. To have bulkheads forwards and backwards… To think less about worries for the future and self-reproach about the past. That resonates with me… and then the brain works with it while I read.*

(Male, Intervention group)

### Resignation and powerlessness

All participants talked about the experience of being ill. Most of them described experiences of psychosis, explaining how emotions were experienced as bodily phenomena and how the symptoms were placed in their lives. Most of the participants also talked about negative and painful experiences, both with previous and current illness and with previous and current treatment. There seemed, however, to be a difference in the way that participants in the two groups talked about these experiences. Patients in the control group expressed more resignation and passivity, giving the impression that they were stuck in these experiences. They used phrases such as "yielding", "enduring", "resigning", "giving in" and "getting used to". Their descriptions included being powerless when facing their lives and problems, a stronger need for care and being more dependent on health services. These patients gave several examples of experiencing hopelessness and powerlessness. Although there were examples of interviews where feelings of helplessness and resignation were less prominent, the following examples represent very common descriptions for the control group participants:

*P: I have accepted the situation I am in. I think I have resigned a little. I have withdrawn from the things that I will never experience.*

*I: Like what?*

*P: Well… [sighs]… my life situation, for instance. I have for instance not had a partner in years… and it is quite stressful because you get very lonely. And after some time you come to a place where you realize that the hopelessness has come to stay.*

(Male, Control group)

Patients who had a contract for self-referral, on the other hand, discussed courage and strength and talked about conscious choices and opinions. Their descriptions reflected being in a better place in their lives, and they seemed more active and less dependent on others. These patients expressed that they had influence over their overall situation.

*P: Well… how do you move on? It is to work… work with yourself. To locate the problems. Some would say to accept the problems, but that sounds a bit defensive to me. Maybe you get there… when you work with your problems they become more distant. You have to learn to like yourself until you get so sure that you are not put off by any little comment… like I used to be. It was… I had a word that emerged in my head during the last hospitalization: "Show off". That I was a show off. Like I knew it all, and wanted to have a piece of everyone else. But then I asked a friend of mine who was admitted during the same period: Do you think I am a show off? And she laughed a little. That is a way to get your problems more distant. Is it really like this? Is it possible? To be confident… to be sure… that you throw it around instead.*

(Male, Intervention group)

There were also a few examples of patients with a contract who seemed to have achieved some distance from their previous bad experiences, as if they had success in moving on with their lives.

*P: And time heals all wounds. That’s how it is. That’s hard to say when you are ill, but when you are recovering you see that it is a one way street. Even though you might be temporarily dumped or feel bad and upset.*

*I: Like a crisis?*

*P: Yes, the crisis lasts around two hours. It’s like that. I don’t know how other people feel, but when I am ill I feel it could last forever. I am scared that it will be a permanent death row, but it doesn’t. It takes shorter and shorter time before I am well again. And that is reassuring.*

*[…] The problems I have aren’t smaller or steeper, only shorter. It feels like I can put them behind me. Previously I could have days and weeks with the same problems and feel thrown around like a ball. Now it takes three or four hours.*

(Male, Intervention group)

### Being normal and living a normal life

Participants in both groups talked about the importance of having a normal life, doing normal things, and feeling like everyone else. A common theme was being identified with the illness, and many emphasized that being ill was not their sole identity. The patients reflected on their own status as persons with mental illness, expressing a desire for an identity beyond the role of a patient.

*P: I have a diagnosis, and that makes me ill – in medical terms. But it is a difference between that and perceiving yourself as an ill person. Cause I am only a guy. I’m just me.*

*[…] I would like to build a different fundament to base the rest of my life on. To build my whole life on the foundation that I am an ill person in a treatment process… that I have an illness that defines me… I feel that it will be a very unstable life. It will fall apart. So I have decided to find the most stable foundation possible… that’s what I’m trying to do.*

(Male, Control group)

An important difference between the patient groups was that patients with a contract for self-referral had several descriptions which seemed much closer to the ideal of living a normal life and being a normal person. They used active phrases when describing their everyday life, such as "I know", "I will", and "I can". They also acknowledged "the good life" and seemed to experience more closeness to a normal everyday life. Patients who had a contract talked about the normal life with more confidence and seemed more independent of social expectations and acceptable values.

*I: What is the point with having an ambulatory supervisor?*

*P: You press an extra button and get feedback on things that might… that you do well or that people notice… that there are some positive things too. They emphasize it to strengthen your self-confidence. The self-confidence isn’t very strong. You get set back from being admitted. I was away for nine months. I was admitted for nine months. Afterwards you are handicapped. While I was admitted I received my meals every day… some places they helped me get up in the morning. I was helped and supported all the time. And that was difficult to give up. But it is very good to have control over your own life. Cooking and having a nice time… to light candles and… to enjoy living.*

(Female, Intervention group)

As part of a normal life, most participants wanted to study or work, and work was associated with identity and a good life. Many of the patients who received treatment as usual highlighted studies and education as a goal which might be difficult to reach, and some of them expressed a great deal of uncertainty regarding the possibility of coping with work on an everyday basis.

*P: I had my job until I realized that it was enough and then I quit.*

*I: You quit?*

*P: Yes. My contract expired… and at the same time I told them I had to quit.*

*I: How was that for you?*

*P: I stayed on the sofa for a week doing nothing. And then I woke up again. It felt like… like I recharged my strength.*

(Female, Control group)

In contrast, many of the patients who had a contract for self-referral seemed to have progressed beyond having education or work as a distant goal. Several had taken the initiative to commence studies and had concrete plans for completion, and they did not mention having to interrupt the progress of their studies due to illness as a problem. They focused instead on the courage and strength necessary to continue and complete.

*P: I found out that the deadline for applications was expired, but I called today and they told me it was fine after all. I have a week to make an application for a […] degree to finish the last two years… […] If I can manage to work with art in some way, or with doing odd jobs. It is very exciting and I look forward to writing things this week and to look through my previous work.*

(Female, Intervention group)

### Experiences with a contract for self-referral or treatment as usual

Although the patients were not asked directly about their experiences with the intervention (contract for self-referral or treatment as usual) some of the patients raised the subject on their own accord. Patients who had a contract for self-referral expressed a great deal of authority and strength when talking about the contract, their contact with the health services and what they needed in the future. Some said that the contract gave them more influence and that this gave them a sense of control. Some also described that they felt safe since they had the option to self-refer at any time:

*P: I believe in the project with self-referral. It is a pillar… where you know that if something happens which is really bad… You know that you can come here and get a break. It feels safe.*

(Female, Intervention group)

Meanwhile, a few of the patients who did not have a contract for self-referral were very dissatisfied:

*P: I am very disappointed to be in the control-group [receiving treatment as usual]. I am done with the ordinary mental health services. I just want the self-referral inpatient contract.*

(Male, Control group)

## Discussion

### Strengths and limitations

Two researchers with extensive user experience in mental health services participated in different parts of this study. One participated in the data collection and took part in the initial analysis, while the other (DB) participated in the analysis process and in writing the manuscript. User participation during the research process strengthens the authenticity of the results and helps ensure that the users’ voices are heard. Since this study was nested within a randomised controlled trial the interviews had to avoid direct questions about the intervention and focus instead on the participants’ general perceptions of their current situation. This made the data material rich and focused on everyday life, which strengthens the quality of the findings. Although the interviewers were advised to avoid direct questions about the intervention, several of the participants talked about having or not having a contract. Some participants who had been randomised to treatment as usual were disappointed, and the interviewers had to respond to this. This feeling of disappointment may have led to negative expressions from those participants. Effort was made to not lead the participants to overstate the positive experiences with the intervention so as to please the interviewers, but the effectiveness of this effort is unclear.

Although the sample is fair-sized for a qualitative interview study, the results might not be valid for other samples in other areas of health services. The interviews were mostly conducted by researchers who have worked as psychiatric nurses at the hospital. The nurses were chosen as interviewers because they were very competent health professionals and familiar to the patients. They were thus able to ensure that patients were taken good care of if they had adverse reactions during or after the interview. In addition, some participants stated that they would not have participated in interviews with unfamiliar interviewers. However, this familiarity might also have led to more favourable evaluations of the hospital’s services and the intervention than if the interviews had been conducted by independent researchers. Moreover, since the participants in the two different groups were treated in the same health service organisation, they had the possibility to interact and discuss their experiences.

Making comparisons based on qualitative interview data is a challenging undertaking. Since the aim of the study emerged during an initial reading of the transcripts, across group allocation, the authors were not "blind" before the more "comparative" analysis. We consider it a strength to pursue the initial surprising findings in the data material. However, at that point we should have included new and "blind" authors in the following analysis, as that would have strengthened the analysis process and the results. We analysed the interviews with patients in the control group before comparing the main themes from the control patients with the interviews with those of the intervention group. The differences between the groups were cross-checked with the data material and discussed in the analysis group to reduce the risk of overstating the differences. The differences presented in the results section are based on general impressions when reading and analysing the interview transcripts and must be interpreted with caution. Considering that several specific words and phrases were more present in either of the two groups, we could have used a computer-based system to compare the occurrences, which could have in turn supported the findings.

Not all participants in the RCT participated in interviews after four months. Despite the initial randomisation, those agreeing to participate in interviews were self-selected. Direct comparison is therefore difficult. In addition, it is not clear whether the differences were due to the intervention (self-referral to treatment) or to other factors. A comparison of the groups on demographic variables (Table 1) also shows that there are some differences between the groups: the participants in the control group were younger, had a higher rate of psychosis diagnosis, and had a higher proportion of males. The differences between the groups in terms of age, diagnosis and gender may have influenced the results of this study.

### Discussion

The results showed that many of the patients with a contract for self-referral expressed more confidence in strategies to cope with mental illness, and they seemed to apply more active cognitive strategies. Patients with a contract also expressed less resignation, hopelessness and powerlessness than patients without a contract, appearing to be closer to the ideal of living a "normal" life and being a "normal" person.

Coping strategies are important in the management of mental illnesses [15, 16]. Such strategies are used to cope with life stressors, to ameliorate symptoms and to prevent periods of symptom aggravation. Most persons who suffer from psychosis apply one or several strategies to cope with mental symptoms [17]. Although there is little knowledge about the usefulness of specific coping strategies, research shows that having several strategies at hand is more effective than relying on just one, although there is not one specific coping strategy which is more effective than the others [17]. In the present study, the patients who had a contract for self-referral described coping strategies which appeared more advanced, especially the cognitive strategies they used to influence their thinking patterns. Strategies to manage mental health symptoms are often taught by professionals during treatment [18]. In addition to these, most persons suffering from mental health illnesses develop personal coping strategies [18]. In the present study, all patients described coping strategies that they had learned, and some of the patients described self-developed strategies. Compared to patients who received treatment as usual, patients with a contract for self-referral described new and different coping mechanisms. Previous evaluations of self-referral have shown that although the total frequency of hospitalizations increased, the number of hospitalization days in fact decreased by more than 30%. Furthermore, the patients reported that a contract for self-referral lessened the burden of having to "convince" professionals that hospitalization was necessary and to be "sick enough" to need inpatient treatment [11, 13]. In other evaluations, patients have also expressed that the opportunity to self-refer gave them safety and confidence to make new efforts and to challenge old behavioral patterns [12]. This might help account for the differences in coping strategies we found in the present study.

Using coping strategies is in line with the recovery model. According to this model, a person with long-term mental illness takes an increasingly active role in managing the impact of the mental illness as they move towards recovery [19]. Coping strategies can be divided into reactive, anticipatory, preventive and proactive strategies [16], and can be placed on a recovery continuum, where proactive strategies are used by persons who have come farthest in the recovery process. One of the findings in the present study was that patients with a contract for self-referral described more confidence in applying coping strategies than patients without a contract. This indicates that the patients with a contract had more experience in successfully using coping strategies and thus had come further in the recovery process. They talked more about being active, growing, breaking barriers and taking advantage of opportunities. This finding is in line with previous research, where empowerment has been described as the most important factor for recovery [18]. The patients who had a contract for self-referral also described some coping strategies which sometimes ran counter to the advice typically given by professionals. This indicates that patients who have had the opportunity to self-refer to treatment felt more in control and more able to contradict or neglect advice from the mental health services. This might be due to the opportunity to be in charge of decisions regarding treatment which could increase autonomy and the feeling of being in control.

In the present study, the participants contrasted the identity of being an ill person with that of being a normal person. The term "illness identity" has been described as a set of roles and attitudes that persons with a mental illness have developed [2]. The participants in the present study related the perception of being a person with an illness to the possibility of being a normal person capable of leading a normal life. Perceiving oneself as an ill person is closely linked to feelings of stigmatization, which is connected to low self-esteem and low self-efficacy [20, 21]. The perception of an illness identity has been described as an important part of the recovery process [2]. Some have linked illness identity to hope and self-esteem, which is in turn linked to coping, engagement, and social interaction [2]. Coping, engagement and social interaction subsequently have an impact on vocational outcomes and symptom severity. Authors have argued that changing illness identity is an important step in improving mental health illness and functioning [2]. The results from the present study indicate that patients with a contract for self-referral might have a more clarified relationship towards their illness identity than patients without a contract, therefore suggesting that the former group may have come further in the recovery process. That said, it is not self-evident that having a contract for self-referral to treatment necessarily impacts illness identity, especially since the present study is based on interviews only four months after signing a contract for self-referral. It would be important to further investigate whether an opportunity to self-refer leads to increased autonomy, sense of control and decision-making power, all of which might subsequently impact the perception of illness identity.

Another important finding in the present study was that the patients who had a contract for self-referral tended to express less powerlessness, resignation and hopelessness than those who received treatment as usual. The patients who received treatment as usual referred to their problems as something that they were stuck with, and they described themselves as being dependent on care from the health services. This difference in focus might be related to the finding that patients in the intervention group had stronger confidence in using coping strategies. Some have argued that stronger hope and self-esteem might lead to less avoidance, more problem-oriented strategies and more social interaction [2]. Previous research has shown that persons with more hope and insight in their illness use more problem-oriented and less avoidant coping strategies [22] and that poor confidence is connected to using avoidant strategies [23]. These findings are in line with the present study, which also illustrates Bandura’s idea of self-efficacy [24]. Self-efficacy is explained as a personal capability and distinguishes one’s belief in capability from self-esteem and whether you like yourself [25].

In the present study, we found that patients with a contract for self-referral talked more about living a normal life with work and studies. They seemed to have a closer relationship to what they called a "normal life" and a somewhat more active attitude towards coping, thereby possibly exhibiting a greater sense of control. Davidson and Roe argue that recovery has to be pursued actively by the patient, and that not everybody does this [3]. The findings in the present study indicate that patients who have had the opportunity to decide whether and when they needed referral to inpatient treatment are more actively pursuing recovery, both *from* and *in* mental illness. In this context signing a contract for self-referral seems to be a rehabilitation approach that encourages and supports activity, self-determination, control, and participation.

## Conclusion

Patients with a contract for self-referral to treatment appeared to have a wider range of coping mechanisms, more self-developed and advanced strategies, and stronger confidence in applying the strategies. The findings indicate that having the opportunity to self-refer might help strengthen autonomy, sense of control, and self-determination. According to research on coping strategies, these patients could be better off both during treatment and in the recovery process. In a similar vein, patients with a contract voiced less resignation, hopelessness and powerlessness and appeared to be closer to experiencing a "normal" life.

## Electronic supplementary material

Additional file 1: **Interview guide - Self-referral inpatient treatment.** (DOCX 29 KB)

## References

[1] Norwegian Ministry of Health and Care Services (2009). Report No. 47 to the Storting 2008-2009 The Coordination Reform.

[2] Yanos PT, Roe D, Lysaker PH (2010). The impact of illness identity on recovery from severe mental illness. Am J Psychiatr Rehabil.

[3] Davidson L, Roe D (2007). Recovery from versus recovery in serious mental illness: One strategy for lessening confusion plaguing recovery. J Ment Health.

[4] Storm M, Edwards A (2013). Models of user involvement in the mental health context: intentions and implementation challenges. Psychiatr Q.

[5] Burns T (2010). The rise and fall of assertive community treatment?. Int Rev Psychiatry.

[6] Sjolie H, Karlsson B, Kim HS (2010). Crisis resolution and home treatment: structure, process, and outcome - a literature review. J Psychiatr Ment Health Nurs.

[7] Hultberg K, Karlsson B (2007). [User experiences with crisis intervention at home]. Tidsskr Norsk Psykol.

[8] Connell J, Brazier J, O’Cathain A, Lloyd-Jones M, Paisley S (2012). Quality of life of people with mental health problems: a synthesis of qualitative research. Health Qual Life Outcomes.

[9] Biong S, Ness O, Karlsson B, Borg M, Kim HS (2012). A crisis resolution and home treatment team in Norway: a longitudinal survey study Part 3. Changes in morbidity and clinical problems from admission to discharge. Int J Ment Health Syst.

[10] Rise MB, Westerlund H, Bjorgen D, Steinsbekk A (2014). Safely cared for or empowered in mental health care? Yes, please. Int J Soc Psychiatry.

[11] Stovind H, Hanneborg EM, Ruud t (2012). [More time with user led admissions]. Sykepleien.

[12] Sollied L, Helland B (2010). [User-led treatment admissions. The way to cope with life?].

[13] Heskestad S, Tytlandsvik M (2008). [Patient-guided crisis admissions for severe psychotic conditions]. Tidsskr Nor Laegeforen.

[14] Norwegian Ministry of Health and Care Service (2011). Official Norwegian Report [Increased Self-determination and Legal Protection] NOU 2011: 9.

[15] Yanos PT, West ML, Smith SM (2010). Coping, productive time use, and negative mood among adults with severe mental illness: a daily diary study. Schizophr Res.

[16] Roe D, Yanos PT, Lysaker PH (2006). Coping with psychosis: an integrative developmental framework. J Nerv Ment Dis.

[17] Phillips LJ, Francey SM, Edwards J, McMurray N (2009). Strategies used by psychotic individuals to cope with life stress and symptoms of illness: a systematic review. Anxiety Stress Coping.

[18] Grealish A, Tai S, Hunter A, Morrison AP (2013). Qualitative exploration of empowerment from the perspective of young people with psychosis. Clin Psychol Psychother.

[19] Davidson L, Roe D, Andres-Hyman R, Ridgway P (2010). Applying stages of change models to recovery from serious mental illness: contributions and limitations. Isr J Psychiatry Relat Sci.

[20] Rusch N, Angermeyer MC, Corrigan PW (2005). Mental illness stigma: concepts, consequences, and initiatives to reduce stigma. Eur Psychiatry.

[21] Schmader T, Major B, Eccleston CP, McCoy SK (2001). Devaluing domains in response to threatening intergroup comparisons: perceived legitimacy and the status value asymmetry. J Pers Soc Psychol.

[22] Lysaker PH, Campbell K, Johannesen JK (2005). Hope, awareness of illness, and coping in schizophrenia spectrum disorders: evidence of an interaction. J Nerv Ment Dis.

[23] Cooke M, Peters E, Fannon D, Anilkumar AP, Aasen I, Kuipers E, Kumari V (2007). Insight, distress and coping styles in schizophrenia. Schizophr Res.

[24] Bandura A (1982). Self-efficacy mechanism in human agency. American Psychologist.

[25] Bandura A (1997). Self-Efficacy. The Exercise of Control.

[CR26] The pre-publication history for this paper can be accessed here:http://www.biomedcentral.com/1472-6963/14/347/prepub

