# Supplementary material for: How do patients with severe mental diagnosis cope in everyday life - a qualitative study comparing patients’ experiences of self-referral inpatient treatment with treatment as usual?
Source: BMC Health Serv Res. 2014 Aug 15;14:347. doi: 10.1186/1472-6963-14-347 (PMC4138383; doi:10.1186/1472-6963-14-347)
Supplement: Supplementary file 1 — Additional file 1: Interview guide - Self-referral inpatient treatment. (DOCX 29 KB) [file 12913_2013_3448_MOESM1_ESM.docx]

**Additional file 1 Interview guide - Self-referral inpatient treatment**

1. How is your life at present?
2. Have you had any experiences lately which have influenced your life (included small everyday things)?
3. Have you been admitted to the hospital lately? (Or received help for your mental problems in any other way?)

a. If yes, how long were you admitted? Was the admission voluntary or not? Did you experience any constraint, threats, persuasion or negative influence during the admission? Why were you admitted?

b. If no, have you felt that you should have been admitted or received help in any other way?

1. How has your mental health been lately? Stable or unstable?

a. If stable, what do you think could be the reason for this? (Ex. substance abuse, regular meals and sleep, social contact, contact with health services.)

b. If unstable, how have you managed being in an unstable period? (Ex. contacted other people for help, abused alcohol or other substances, tried to push the thoughts away.)

1. Have you had any changes in your medication lately? (Type of medicine or dosage.)
